# Familial Hemiplegic Migraine with Severe Attacks: A New Report with* ATP1A2* Mutation

**DOI:** 10.1155/2016/3464285

**Published:** 2016-10-13

**Authors:** E. Martínez, R. Moreno, L. López-Mesonero, I. Vidriales, M. Ruiz, A. L. Guerrero, J. J. Tellería

**Affiliations:** ^1^Neurology Department, Hospital Clínico Universitario, Valladolid, Spain; ^2^Clinical Analysis Department, Hospital Clínico Universitario, Valladolid, Spain; ^3^IBGM, University of Valladolid, Valladolid, Spain

## Abstract

*Introduction*. Familial hemiplegic migraine (FHM) is a rare disorder characterized by migraine attacks with motor weakness during the aura phase. Mutations in CACNA1A, ATP1A2, SCN1A, and PRRT2 genes have been described.* Methods*. To describe a mutation in ATP1A2 gene in a FHM case with especially severe and prolonged symptomatology.* Results*. 22-year-old woman was admitted due to migraine-type headache and sudden onset of right-sided weakness and aphasia; she had similar episodes in her childhood. Her mother was diagnosed with hemiplegic migraine without genetic confirmation. She presented with fever, decreased consciousness, left gaze preference, mixed aphasia, right facial palsy, right hemiplegia, and left crural paresis. Computed tomography (CT) showed no lesion and CT perfusion study evidenced oligohemia in left hemisphere. A normal brain magnetic resonance (MR) was obtained. Impaired consciousness and dysphasia began to improve three days after admission and mild dysphasia and right hemiparesis lasted for 10 days. No recurrences were reported during a follow-up of two years. We identified a variant in heterozygous state in ATP1A2 gene (p.Thr364Met), pathogenic according to different prediction algorithms (SIFT, PolyPhen2, MutationTaster, and Condel).* Conclusion*. Prolonged and severe attacks with diffuse hypoperfusion in a FHM seemed to be specially related to ATP1A2 mutations, and p.T364M should be considered.

## 1. Introduction

Familial hemiplegic migraine (FHM) is an uncommon type of migraine with aura including motor weakness with at least one first- or second-degree relative affected [1]. FHM is considered a monogenic disorder with autosomal dominant inheritance pattern. Though there may be other loci to be identified, FHM is subdivided into three types: FHM1 with mutations in* CACNA1A *gene on chromosome 19, FHM2 in* ATP1A2 *gene on chromosome 1, and FHM3 in which mutations in* SCN1A *gene on chromosome 2 have been identified [1]. These genes are implicated not only in ion channel but also in other molecules as synaptosomal associated protein. Moreover, there are typical cases that do not have a mutation in one of the main genes described, suggesting that other genes are still to be identified [2].

The clinical spectrum of this disorder varies from moderate headache accompanied by motor weakness to coma, with description of cases associated with permanent ataxia, epileptic seizures, mental retardation, and chronic progressive cerebellar atrophy [2].

We describe a mutation in* ATP1A2 *gene in a case of FHM with especially severe attacks.

## 2. Case Report

A 22-year-old Caucasian woman was admitted due to a clinical picture of right-sided weakness and aphasia accompanied by a migraine-type headache (throbbing pain on the left side, with nausea and photophobia) initiated when she woke up. She had previously suffered similar clinical episodes 7 years before and during her childhood. Her mother presented with multiple episodes of migraine with motor weakness. Our patient was adopted during early childhood and did not have a relationship with her family; so, a more accurate description on her mother's clinical picture was not available. She has no brothers or sisters.

In the anamnesis of possible triggers, she reported that, ten days before admission, she had suffered a mild head trauma during a traffic collision (she did not call for medical care for this reason). At admission, clinical exam showed a left gaze preference, predominantly nonfluent aphasia, and right facial palsy with right hemiplegia.

**Figure 1:**
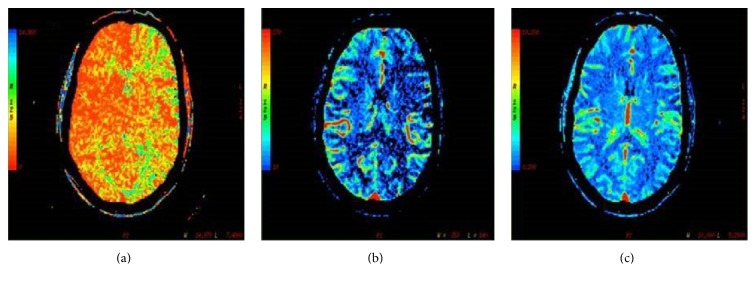
Perfusion computed tomography. TC revealed increased mean transit time (a) and diminished cerebral blood flow (b) with preserved cerebral volume (c). These changes were observed throughout the entire left cerebral hemisphere not confined to a particular vascular territory including the territory of anterior, middle, and posterior cerebral arteries (PCA).

Urgent unenhanced CT was normal, but perfusion CT revealed increased mean transit time (MTT) and diminished cerebral blood flow (CBF) throughout the entire left cerebral hemisphere not confined to a particular vascular territory including the territory of anterior (ACA), middle (MCA), and posterior cerebral arteries (PCA) (Figure 1). Cerebral blood volume (CBV) was normal. Findings were consistent with hypoperfusion throughout all the left cerebral hemisphere. Cerebrovascular study was completed with an angiography TC and carotid ultrasound with no alterations. Transcranial Duplex revealed a generalized acceleration in both middle cerebral arteries with absence of arterial occlusion. A diffusion-weighted brain magnetic resonance imaging (DWB-MRI) was performed 3 days after clinical onset and it showed no restricted diffusion on the region of perfusion abnormality with normal signal intensity of the brain parenchyma.

On the second day, aphasia worsened, and somnolence and body temperature of 38° appeared. A lumbar puncture was performed without pleocytosis nor hyperproteinorrachia. Cerebrospinal fluid (CSF) microbiological studies and serum extensive biochemical, hematological, or immunological determinations were carried out with normal results. An electroencephalogram (EEG) recording showed diffuse delta activity.

The patient was empirically treated with acetylsalicylic acid and acyclovir during the first days. The clinical picture evolved favorably but in a slow way; fever and diminished level of consciousness disappeared on day 4, and motor deficit and dysphasia resolved, respectively, 6 and 8 days after onset. No recurrence of neurological symptoms was observed during a follow-up of 2 years.

As usual in our protocols for FHM studies, we carried out exome sequencing and subsequently analyzed the coding sequences of* SCN1A, CACNA1A, ATP1A2, NOTCH3*, and* PRRT2 *genes in order to identify potential pathogenic mutations. DNA was extracted from peripheral blood according to standard protocols. DNA quality was determined by continuous reading of optical density with the Nanodrop equipment. Its integrity was checked by electrophoresis and SYBR Green II staining. After checking the quality, we proceeded to the capture and massive sequencing of human gene exons by using the SureSelect Human All Exon Kit 51 Mb (Agilent®) assay and Hiseq2000 (Illumina®) sequencer with a 30x mean coverage depth. The bioinformatic analysis allowed us to identify single nucleotide variants and small insertions and deletions relative to the reference genome [25]. Once obtained, the exonic sequences were aligned against the human genome reference, and we proceeded to the selective detection of all possible variants in candidate genes. Variants reported with frequencies over 1% in dbSNP, 1000 Genomes, and Exome Variant Server were excluded and the results were analyzed assuming a dominant model of inheritance according to the model expected for the disease and the indicated genes.

We identified a heterozygous variant within* ATP1A2 *gene. This variant has not been reported in more than 6500 control individuals [26]. Sequence analysis revealed C>T transition (missense mutation) in position 160098515 of chromosome 1. This variation in* ATP1A2 *gene produces Thr to Met amino acid substitution at position 364 of the coded protein (p.T364M).

The substituted amino acid is strongly conserved, since it is present in homologous proteins in all recorded mammals, reptiles, and fishes. Several in silico tests strongly suggest that Thr to Met substitution should strongly affect the* ATP1A2 *function: PolyPhen shows a damaging score of 1.000 and reports that Thr is always conserved in this position when compared with the homologous gene of 43 species; MutationTaster software test gives a probability of disease causing over 0.9999 and reports that this mutation has not been reported in 1000G or in ExAC databases; and, finally, SITF test gives a PROVEAN score of −5.166 (variants with scores under −2.5 are considered deleterious).

No further potential pathogenic mutations were found within* ATP1A2 *or in the remaining candidate genes studied. The variant was validated by Sanger sequencing.

## 3. Discussion

FHM is a clinically heterogeneous disorder, with attacks varying from mild hemiparesis to severe long-lasting hemiplegia. Rare disturbances of consciousness (sometimes including coma), confusion, fever, and cerebrospinal fluid pleocytosis may occur [2].

There are more than 60 different missense mutations in* ATP1A2 *gene. Other heterozygous mutations in* ATP1A2 *gene have been reported in FHM patients [3–6, 9, 18]. We present a table with the mutations described in the literature from 2003 to 2016 and their main phenotypic characteristics (Table 1). Some of the mutations produce long-lasting attacks of hemiplegic migraine, but they are related to other neurological pathologies such as seizures and mental retardation [1], coma, and fever [23]. The case we present had no other neurological diseases apart from FHM.

To understand the relationship of the ATP1A2 gene with seizures, migraine, and coma, it is important to understand the pathophysiology of the mutations in ATP1A2 gene. This gene is on chromosome 1q23 and encodes *α*-2 subunit of Na^+^/K^+^ ATPase plasma membrane enzyme, which consumes ATP to actively transport Na^+^ out of the cell and K^+^ into the cell [9]. Na^+^/K^+^ ATPase protein is composed of three heteromeric subunits (*α*, *β*, and *γ*), and *α* is the catalytic one, which is composed of two subunits. The *α*-2 subunit is expressed in central nervous tissue, particularly astrocytes and pyramidal cells in the hippocampus. This *α*-2 subunit is composed of N-terminal region containing 4 membrane-spanning domains (M1–M4) and C-terminal region containing 6 membrane-spanning domains (M5–M10), which are linked by a large intracellular loop. This large M4-M5 loop contains critical functional sites and undergoes major conformational changes during the enzymatic cycle. Most of the* ATP1A2 *gene mutations causing FHM2 are located within this loop [9]. In fact, the described mutation affects the M4-M5 loop. In silico software predicts severe conformational changes within this domain on the mutated protein. Moreover, Thr amino acid is highly conserved underlining its key role in this location of ATP1A2 protein. This variation in* ATP1A2 *gene produces Thr to Met amino acid substitution at position 364 of the coded protein (p.T364M), leading to the dysfunction of the gene.

As Na^+^/K^+^ ATPase exchanges intracellular Na^+^ for extracellular K^+^, loss of Na^+^/K^+^ ATPase function results in raised extracellular K^+^, which facilitates cortical spreading depression, the mechanism postulated to cause migraine aura. This ATPase dysfunction also results in raised intracellular Na^+^, which will increase intracellular Ca^2+^ levels as a result of a decrease in Na^+^/Ca^2+^ exchange. This raised intracellular Ca^2+^ also facilitates the cortical spreading depression [9] with the consequent hypoperfusion as we have shown in Figure 1. These biochemical alterations are capable of producing prolonged functional impairment but without causing lesion on neuroimaging (MRI).

The mutation present in our patient was previously described in an eight-year-old female who presented with prolonged attacks including hemiplegia, aphasia, lethargy, and fever [18]. As in our case, cerebrospinal fluid was unremarkable, and electroencephalogram showed diffuse slowing. Hypoperfusion was not shown in perfusion CT as in our patient.

## 4. Conclusion

In conclusion, prolonged and severe attacks with diffuse hypoperfusion in a FHM seemed to be specially related to* ATP1A2 *mutations, and p.T364M mutation should be considered.

## Figures and Tables

**Table 1:** Mutations described in *ATP1A2 *gene related to FHM.

Mutation	Phenotypic characteristics
*D718N [1]*	Long-lasting hemiplegic migraine, seizures, and mental retardation
*P979L [1]*	Recurrent coma, seizures, mental retardation, and interictal mild cerebellar signs
*R689Q [2]*	Benign familial infantile convulsions
*K7940 [2]*	Spectrum between alternating hemiplegia of childhood (AHC) and FHM
*A1033G, T345A [3]*	Coma
*E700K [2]*	FHM
*R593W and V628M [4]*	FHM
*M731T and T376M [5]*	Pure FHM
*A606T, N717K, R1002Q [6]*	Severe hemiplegia
*T415M [7]*	Dysphasia and drowsiness and attacks triggered by mild head injury
*V362E [8]*	Mood alterations, classified as a borderline personality
*P796S [8]*	Mild mental impairment, in addition to hemiplegic migraine
*D999H [8, 9]*	Seizures
*G900R [8]*	Coma, high fever, and status epilepticus
*G301R [10, 11]*	FHM with interictal cerebellar symptoms
*R548C [12]*	Epilepsy
*G855R [13]*	Febrile seizures
*G902L [14]*	Fever, coma, and cortical edema in MR
*V338A, Q927P [11]*	Coma
*G715R [15]*	Aphasia, coma, and brain edema
*R548H [16]*	Hemiplegic migraine associated with basilar migraine
*M731T [17]*	Psychotic aura symptoms
*p.T364M [18]*	Prolonged hemiplegia, aphasia, somnolence, and fever in a child
*R1007W [19]*	Drowsiness with myoclonic seizures
*S220L, R908Q [20]*	Coma and aphasia
*M731T [21]*	Psychotic aura
*R908Q [22]*	FHM with prolonged aura
*p.A348p [23]*	Large family and severe and long-lasting attacks with coma and fever
*c.G571A [24]*	Neurosensorial hearing loss
